# Chromosomal Translocations Detection in Cancer Cells Using Chromosomal Conformation Capture Data

**DOI:** 10.3390/genes13071170

**Published:** 2022-06-29

**Authors:** Muhammad Muzammal Adeel, Khaista Rehman, Yan Zhang, Yibeltal Arega, Guoliang Li

**Affiliations:** 1 National Key Laboratory of Crop Genetic Improvement, Huazhong Agricultural University, Wuhan 430070, China; mmadeel@webmail.hzau.edu.cn (M.M.A.); zhangyan4963@sina.cn (Y.Z.); 2 Agricultural Bioinformatics Key Laboratory of Hubei Province, Hubei Engineering Technology Research Center of Agricultural Big Data, 3D Genomics Research Center, College of Informatics, Huazhong Agricultural University, Wuhan 430070, China; 3 Department of Environmental Health Science, College of Public Health, University of Georgia, Athens, GA 30605, USA; 4 State Key Laboratory of Agricultural Microbiology, Huazhong Agricultural University, Wuhan 430070, China; khaistabiotech@webmail.hzau.edu.cn; 5 College of Veterinary Medicine, Huazhong Agricultural University, Wuhan 430070, China; 6 College of Bio-Medicine and Health, Huazhong Agricultural University, Wuhan 430070, China; 7 Departments of Population Health & Environmental Medicine, New York University 180 Madison Ave., New York, NY 10016, USA; yabyibe@gmail.com

**Keywords:** 3D-genome, translocations detection, cancers, WGS, PCR, Hi-C, bioinformatics

## Abstract

Complex chromosomal rearrangements such as translocations play a critical role in oncogenesis. Translocation detection is vital to decipher their biological role in activating cancer-associated mechanisms. High-throughput chromosomal conformations capture (Hi-C) data have shown promising progress in unveiling the genome variations in a disease condition. Until now, multiple structural data (Hi-C)-based methods are available that can detect translocations in cancer genomes. However, the consistency and specificity of Hi-C-based translocation results still need to be validated with conventional methods. This study used Hi-C data of cancerous cell lines, namely lung cancer (A549), Chronic Myelogenous Leukemia (K562), and Acute Monocytic Leukemia (THP-1), to detect the translocations. The results were cross-validated through whole-genome sequencing (WGS) and paired-read analysis. Moreover, PCR amplification validated the presence of translocated reads in different chromosomes. By integrating different data types, we showed that the results of Hi-C data are as reliable as WGS and can be utilized as an assistive method for detecting translocations in the diseased genome. Our findings support the utility of Hi-C technology to detect the translocations and study their effects on the three-dimensional architecture of the genome in cancer condition.

## 1. Introduction

Human genome continuously undergoes genetic changes to a different extent, and about 0.6% of the normal genome is under continuous genomic variations, including translocations, inversions, deletions, and duplication pressure. The size of genetic variations can be minor such as single nucleotide polymorphisms (SNPs), to larger (>50 bp), including all types of structural variations (SVs). These SVs are among the primary reasons behind the differential genetic makeup among individuals as well as normal to diseased genomes [1,2]. Structural variations, specifically chromosomal translocations (balance and imbalance), occur due to inter-chromosomal segment exchange and cause genome rearrangements. These genomic rearrangements give rise to several abnormalities within the genome and cause abnormal phenotypic expressions [3]. In cancer, SVs are reported as the driving force behind oncogenesis, either by disrupting regulation or changing gene dosage [4]. Some germline SVs (whole-chromosome abnormalities or microdeletion and microduplication syndromes) have been reported as a pathogenicity factor of congenital disorders [5,6]. Structural variations have a high chance of affecting the cis-regulatory elements within the non-coding regions by changing their positions or functions. These disrupted cis-regulatory elements show loss of enhancer–promoter interactions (E–P) and lead to the misregulation of genes. Three-dimensional (3D) genome studies have revealed that the position effects of SVs cannot merely be determined by the linear alterations but also by the spatial arrangement of the genome [7].

High-throughput sequencing methods have greatly progressed in detecting and characterizing clinically significant single nucleotide polymorphisms (SNPs) and complex rearrangements. In the post-genomic era, several sequence-based technologies have been devised to detect SVs. Currently, whole-genome sequencing (WGS) and Genome-wide association study (GWAS) technology have shown promising throughput for variants detection [8,9]. However, some limitations are associated with the utility of these technologies, for example, high-false positives rate, dependency of results on reads-length (short and long reads), and deficit of power to detect complex chromosomal rearrangements [10,11,12,13]. Moreover, sequence-based techniques cannot explain the consequences of variations on the three-dimensional geometry of genomes. Thus, the complete mechanism of disease development cannot be fully unveiled.

Comparatively, high-throughput chromosomal conformation capture (Hi-C) has been considered the most valuable source for detecting complex rearrangements such as chromosomal translocations because it can detect balance variations (copy number neutral events) [14,15]. The detection of translocations is vital to decipher their role in gene regulations and pathogenicity. Several Hi-C-based tools are publicly available for detecting translocations, but the validation of their results is yet to be explored.

In this study, we have used high-throughput chromosomal conformation capture (Hi-C), and whole-genome sequence (WGS) data from three different cell lines, namely lung cancer (A549), chronic myelogenous leukemia (K562), and acute monocytic leukemia (THP-1) cell line. Hi-C technology-based tools were applied to detect translocations, compared, and cross-validated with WGS results. Moreover, the precise locations of breakpoints were amplified through the PCR method. Our data suggested the presence of high-resolution translocation breakpoints; additionally, we proposed that besides WGS, Hi-C data can be applied as an assistive method for detecting clinically significant translocations in cancer genomes.

## 2. Materials and Methods

### 2.1. Data Source

In this study, we have used the data of three cell lines, lung cancer (A549), chronic myelogenous leukemia (K562), and acute monocytic leukemia (THP-1), for the analyses. Two kinds of high-throughput chromosomal capture data (Hi-C), i.e., Digestion Ligation Only (DLO) Hi-C [16] and in situ Hi-C [17], and whole-genome sequence (WGS) data were retrieved from Sequence Read Archive (SRA) (https://www.ncbi.nlm.nih.gov/sra, accessed on 13 February 2021) of NCBI (Table 1).

### 2.2. Hi-C Data Processing

Quality control analysis of Hi-C fastq reads was performed by FastQC v0.11.8 [18]. The adapters and linkers were removed using the appropriate clipper in Trimmomatic [19]. Refined reads were subjected to Hi-C processing using two independent pipelines, i.e., DLO Hi-C tools [20] and HiC-Pro [21], depending upon the Hi-C experiment. DLO Hi-C data of K562 and THP-1 were processed through the DLO Hi-C tools, followed by steps such as (1) pre-processing, (2) read alignment and filtering, (3) noise reduction and paired-end reads classification, and (4) interaction visualization [20]. Lung cancer (A549) cell line data were generated by an in situ Hi-C experiment and processed through HiC-Pro [21]. Contact matrices were developed at different resolutions and normalized using the ICE normalization method [22].

### 2.3. Translocations Detection

Recently, several tools have been published for the detection of translocations using Hi-C as input data. Previously, several traditional techniques such as PCR, Southern blotting, and fluorescent in situ hybridization (FISH) have been applied to detect genomic rearrangements. These methods have very low throughput, or cannot precisely locate the breakpoints of rearrangements [23,24]. HiC_trans is the first designed tool based on Hi-C data to detect translocations. HiC_trans takes pre-processed HiC data in fixed matrix form (usually 40 kb for human genome) and scans inter-chromosomal interactions based on change-points [14]. In our analysis, Hi-C matrix data of A549, K562, and THP-1 cell lines at 40 kb resolutions were generated using independent modules of HiC-Pro and used as an input for HiC_Trans. Translocations were detected using default parameters, and multi-resolution breakpoints were detected and used for downstream analyses. Another most useful tool hic_breakfinder (https://github.com/dixonlab/hic_breakfinder, accessed on 18 February 2021) was used for the detection of translocations in the above-mentioned cell line data. Compared to the HiC_Trans, hic_breakfinder uses aligned file, and inter- and intra-chromosomal expectations file of the human genome assembly as an input. Low-quality reads from mapped input files were removed, and the highest resolution (1-kb) parameter was selected for the final results. Additionally, multiple intermediate resolution outputs were generated, and optimal translocations were refined and used in subsequent steps. HiNT-TL was applied as a third tool for translocations detection [25]. Unique and overlapped breakpoints were detected by using bedtools pairToPair tool. Hi-C maps were visualized by juicebox [26].

### 2.4. Whole-Genome Sequence Processing

The quality control analyses of A549, K562, and THP-1 WGS data were performed by FastQC [18] and Trimmomatic [19], reads were aligned against the reference genome (hg19 assembly) using Burrows-Wheeler Alignment (BWA) tool [27] at default parameters, and the duplicates were marked and removed using Picard (http://broadinstitute.github.io/picard/, accessed on 19 May 2021). SAMtools was used for alignment quality estimation and sorting bam reads [28]. For structural variations (SVs) detection in the WGS data of the given cell lines, we used Manta-tumor only SV caller at default parameters. Manta tool accurately discovers variants of small as well as large-sized indels and translocations in a parallel manner [29]. Additional refinement “PASS” parameter was applied, and results were visualized by Integrative Genome Viewer (IGV) [30].

### 2.5. PCR Validation

Optimal breakpoints genomic coordinates from Hi-C data were selected for each sample, and PCR primers were designed using primer3 suite (https://primer3.ut.ee/, accessed on 16 November 2021). PCR amplifications were performed using KAPA HiFi HotStart ReadyMix (07145446001), electrophoresed on agarose low melting gel with DNA Marker as a ladder.

## 3. Results 

**Figure 1:**
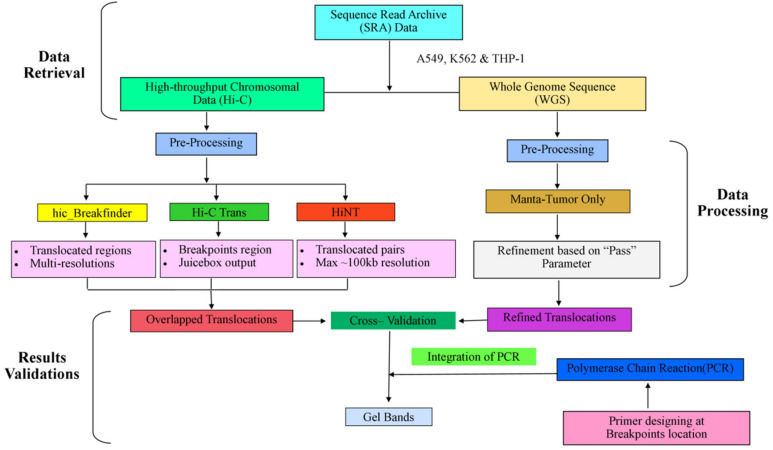
The complete workflow of data analysis.

The overall summary of the methodology has been described in Figure 1, which is divided into three major parts (1) data retrieval, (2) data processing, and (3) validation of results.

### 3.1. 3D-Genome Variations across Different Cell Lines

Genome-wide Hi-C map analyses of A549, K562, and THP-1 cell lines were performed. We compared each cell with normal cell line GM12878 Hi-C data [17] to find differential 3D-genome rearrangements. For the A549 cell line, two replicates of in situ Hi-C were used with 1,075,376,366 and 909,248,853 valid read pairs. In K562 cell line data processing, three replicates (SRR6468005, SRR6468006, and SRR6468007) of DLO Hi-C data were analyzed and combined in subsequent steps. A total of 662,393,544 reads were refined. In the linker’s filtering step, 581,910,908 AA linkers, 74,299 AB linkers, 72,536 BA linkers, 55,636 BB linkers, and 78,914,392 ambiguous linkers were detected. DLO Hi-C of THP-1 (SRR5005053) was analyzed through the DLO-HiC tool, a total of 127,053,079 reads were used for downstream analyses. In linker’s filtering step 40,973,662 AA linkers, 2,310,339 AB linkers, 2,664,559 BA linkers, 46,915,203 BB linkers, and 34,151,836 ambiguous linkers were filtered out.

**Figure 2:**
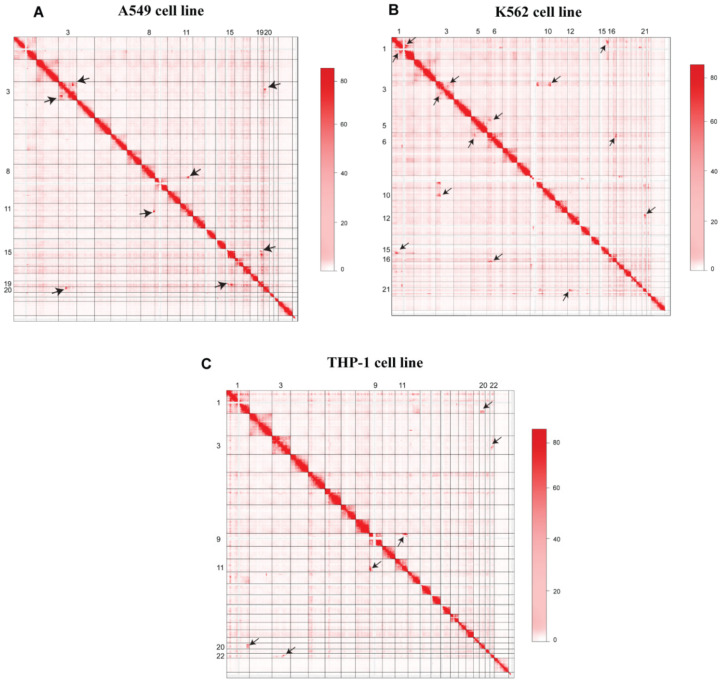
Hi-C maps of A549, K562, and THP-1 3D-genome. (**A**) Heatmap showing the chromosomal interaction in A549 cell line, (**B**) Contact matrix representing the 3D-Genome of K562 cell line, and (**C**) Heatmap representing THP-1 cell line 3D-genome. Different rearrangements were observed and marked with black-headed arrows.

**Figure 3:**
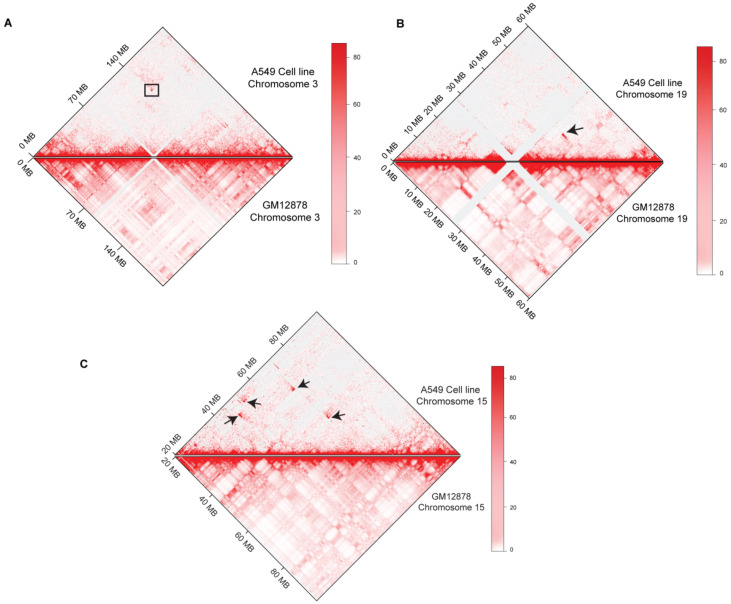
Differential Rearrangements at the chromosomal level in A549 cell line. Comparison between A549-Cell line (Upper panel) and Normal GM12878 (Lower panel) Hi-C at chromosomal level is shown (**A**) chromosome 3, (**B**) Chromosome 15, and (**C**) Chromosome 19. Changes are marked with black arrows and box.

A visual inspection of Hi-C maps showed several higher-order genomic rearrangements at a genome-wide level (Figure 2). Four rearrangements were observed in the A549 cell line, seven rearrangements were observed in the K562 Hi-C map, and three dis-organizations were observed in the THP-1 cell line. Disorganized regions on Hi-C maps were highlighted with the black arrowheads. We also observed the 3D-genome changes at the chromosomal level. In A549 cell line, pre-processed GM12878 Hi-C data were used as a control to find differential chromatin interactions at the chromosomal level. Juicebox was used to visualize the differential architecture of the chromosomes. Most of the chromosomes showed consistent organization between normal GM12878 and A549 cell line Hi-C data. However, chromosome 3, chromosome 15, and chromosome 19 showed higher-order rearrangements (Figure 3). A reorganization was observed in chromosome 3 at ~140 Mb region in A549 cell line, marked with the black box, and it was missing at the corresponding region in GM12878. In chromosome 15, several differential rearrangements were observed in the genomic region between 40 and 80 Mb. Moreover, we observed disorganization at ~45 Mb region of chromosome 19, marked with the black arrowhead.

In the K562 cell line, compared with GM127878, we observed some disorganizations in several chromosomes, such as in chromosomes 6, 10, 15, and 16. Genomic regions with differential organizations were highlighted with the dotted and green boxes. In chromosome 6, a region from 0 Mb to ~150 Mb showed variable structure highlighted with a spotted box. In Chromosome 10, a genomic region from 67.75 Mb to 135 Mb showed variability; two strip-like structures were missing in the K562 cell line. Similarly, in chromosomes 15 and 16, several reorganizations were observed and marked by dotted black boxes (Supplementary Figure S1).

In THP-1 cell line, chromosomes 1, 9, and 20 were among the chromosomes that underwent higher-order three-dimensional structural changes. For example, in chromosome 1, a few variations were observed at ~90 Mb, ~110 Mb, 200 Mb, and 220 Mb regions marked by arrowheads. In chromosome 9, a genomic region from ~10 Mb to 70 Mb, and ~135 Mb region showed the highest variability compared with the GM12878 cell line shown with dotted box and arrowhead. Most of the variations in both 1 and 9 chromosomes have occurred away from the diagonal (Supplementary Figure S2).

### 3.2. High-Resolution Translocations Detection

**Figure 4:**
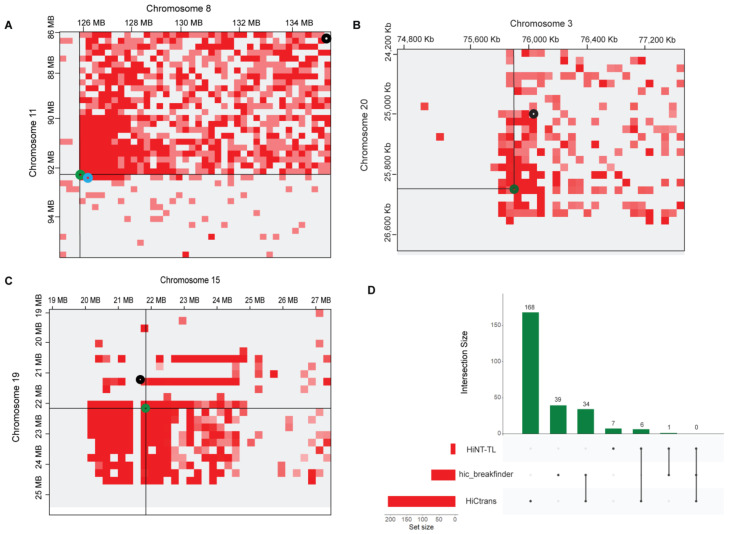
Translocations identified in A549 cell line in magnified representation of translocated regions detected by Hi-C-based tools. (**A**) breakpoints between chromosome 8 and chromosome 11, (**B**) breakpoints between chromosome 3 and chromosome 20, and (**C**) breakpoints between chromosome 15 and chromosome 19. Green circle; HiC_trans, Black circle; hic_breakfinder and Cyan circle HiNT-TL. (**D**) UpSet plot is showing the breakpoints results of translocations detected by different methods. Black dots indicating sample set, and vertical black line is showing the intersection between methods.

Higher-order rearrangements were previously assigned as “structural variations” such as translocations, insertions, and deletions [7,15,31]. To precisely nominate these higher-order rearrangements as the translocations, we processed all cell lines Hi-C data using hic_breakfinder [31], HiC_trans [14], and Hi-C for copy Number variation and Translocation detection (HiNT) [25]. In A549 cell line data, HiC_trans collectively detected 168 breakpoints not only in three sets of translocated chromosomal pairs (3–20, 8–11, and 15–19) but also in additional chromosomal pairs. The 40 kb resolution matrices were used as input in HiC_trans as recommended (Supplementary Table S1). Hic_breakfinder takes Bam files as input to detect translocations in given data. We provided bam files for each analysis; 39 breakpoints were detected in different chromosomes at multiple resolutions such as 1, 10, 100 Kb, and 1 Mb (Supplementary Table S2). HiNT-TL was also applied for translocation detected, which detected seven breakpoints in different chromosomal pairs, i.e., chr8-chr11, chr15-chr19 and 15,18,3,4 paired with chr21 (Supplementary Table S3). In the chr8-chr11 pair, HiNT-TL predicted breakpoints in the adjacent breakpoint region detected by hic_breakfinder, while in other pairs, either the detected breakpoints were out of frame or were not detected (Figure 4).

To find the consistency of the results among available tools, we compared the results to find unique and overlapped breakpoints. A total of 34 breakpoint regions were found common between hic_breakfinder and HiC_trans, 6 breakpoints were overlapped between HiNT-TL and HiC_trans, and only 1 breakpoint was found overlapping between HiNT-TL and hic_breakfinder; no overlapping results were found in all three tools (Figure 4). Several translocated chromosomal regions in the K562 cell line were also detected, HiC_trans detected a total of 169 breakpoints at 40 kb resolutions. Chromosomal pairs 1-15, chr3-chr10, chr6-chr5, chr12-chr21, and chr6-chr16, were showing prominent translocations (Supplementary Figure S3). Four breakpoints were detected in chr1-chr15, one breakpoint point in chr3-chr10, four in chr5-chr6, seven in chr12-chr21, and five were detected in chr6-ch16 pair (Supplementary Table S1). In K562 cell line, Hic_breakfinder detected 114 translocations between several chromosomal pairs, including chr1-chr15, chr3-chr10, chr6-chr5, chr12-chr21, and chr6-chr16 at 1 Kb, 10 Kb, 100 Kb, and 1 Mb resolutions. One breakpoint was found in chr1-chr15 and chr3-chr10 each. Two breakpoints were detected in chr6-chr16, and a single breakpoint was detected in chr6-chr5 and chr12-chr21 each (Supplementary Figure S3, Table S2). HiNT-TL tool was not applied to detect translocations in K562 because it can only process the Hi-C data with HindIII, DpnII, and MboI restriction enzymes [25]. In contrast, our data were generated with a MseI cutter, thus incompatible with this pipeline. Unique and overlapped breakpoints were detected between HiC_trans and hic_breakfinder. Since HiC_trans detected 169 breakpoints and hic_breakfinder predicted 114 breakpoints, 23 were found common between both these tools (Supplementary Figure S4).

THP-1 cell line Hi-C data were also analyzed by HiC_trans and hic_breakfinder to detect translocations. HiC_trans detected a total of 39 breakpoints genome-wide, chr1-chr20, chr3-chr22, and chr9-chr11. Translocations were prominent in chr9-chr11 and chr3-chr22. Four breakpoints were found in chr9-chr11, and two breakpoints were detected in chr3-chr22. Three breakpoints were found in the chr1-chr20 chromosomal pair, but their locations were found at the false-positive region in the Hi-C map, where no apparent interactions were observed (Supplementary Table S1). Hic_breakfinder detected 35 breakpoints in the THP-1 sample, chr3-chr22 and chr9-chr11 showed two translocations each (Supplementary Table S2). No translocation was detected in chr1-chr20 pair. Results from both tools were compared, and 13 common breakpoint regions were detected (Supplementary Figures S5 and S6).

### 3.3. Cross-Validation of Translocations in WGS Data

WGS data from each sample (A549, K562, and THP-1) was analyzed to detect SVs, including translocations, insertions, and deletions. Another purpose of WGS analysis was to cross-validate the translocations identified in Hi-C data. Manta Tumor-Only Analysis was performed to find the structural variants [29]. Here, we only consider those pairs of chromosomes in which Hi-C-based translocations were found. In A549 WGS data, manta has predicted 22,382 breakpoints, 5287 deletions, 1095 duplications, and 1405 insertions. Out of 22382, 108 breakpoints were found in chr11-chr8, 90 in chr3-chr20, and 72 were in chr15-chr19 chromosomal pairs. In K562 cell line WGS data, 43,444 breakpoints, 5151 deletions, 1277 duplications, and 1340 insertions events were detected genome wide. A total of 320 breakpoints were found in chr1-chr15, 300 in chr3-chr10, 482 in chr5-chr6, 60 in chr12-chr21, and 236 were detected in chr16-chr6 pair. In THP-1 WGS at genome wide, 8800 breakpoints, 4136 deletions, 963 duplications, and 1153 insertions were reported. At the chromosomal level, 30 breakpoints were detected in the chr3-chr22 pair, 126 were in chr9-chr11, and 158 were found in chr1-chr20.

**Figure 5:**
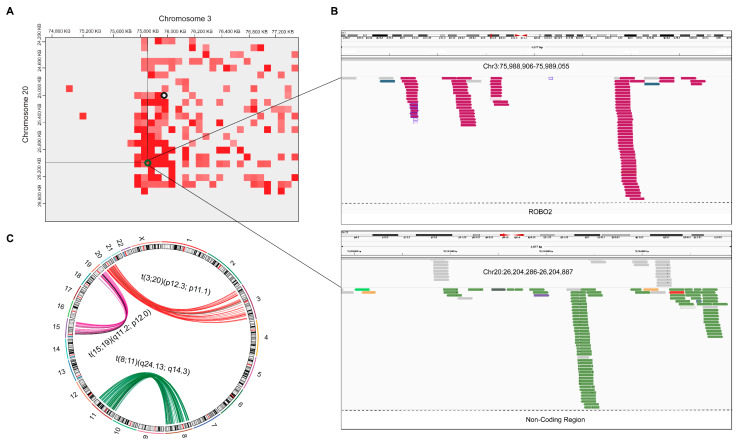
Cross-validation of Hi-C-based translocations with mate pairs analysis in WGS data. (**A**) breakpoints between chromosome 3 and chromosome 20, Green circle; HiC_trans, Black circle; hic_breakfinder. (**B**) In t(3;20)(p12.3;p11.1), mate pairs are shown in upper and lower panel with deep pink and light-green color, respectively. (**C**) In circos plot, translocations detected by Manta in each chromosomal pair are shown with red, purple, and green. Hi-C overlapped results were highlighted with black arcs.

Further, we performed a paired-end analysis to identify the presence of split-pairs and validated the translocation identified from Hi-C data. In A549 cell line, the positions of all translocations were coherent with the Hi-C data. The presence of each paired read was manually inspected through the IGV tool. Similarly, in t(8,11)(q24.13;q14.3), *FAT3* gene was located in translocations at chromosome 11. In t(15;19)(q11.2;p12.0), *ZNF43* gene was detected in the translocation site at chromosome 19, and *NR_110480* novel transcript was detected in chromosome 15 (Supplementary Figure S7). In t(3;20)(p12.3;p11.1), *ROBO2* gene was located at chromosome 3, no gene was present at the corresponding region at chromosome 20. Manta-based results showed the presence of translocations in different sets of chromosomes. Manta results validated Hi-C results and predicted novel breakpoints (Figure 5).

In K562 WGS data, the presence of mate-paired reads validated Hi-C results. In t(15;1)(q24.1;q25.1) and t(12;21)(p12.1;q21.2), no coding gene was detected in corresponding regions. In t(3,10)(p21.33;q23.2), *CDG25A* and *GRID1* genes were located at chromosome 3 and chromosome 10, respectively. In t(16;6)(q21;p21.33), *ITPR3* gene was observed at chromosome 6, while the gene desert region was detected in the corresponding region at chromosome 16 and in t(5;6)(q11.2;p12.1), *GUSBP1* was located at chromosome 5 and gene-desert was observed in chromosome 6. Manta also validated the Hi-C results (Supplementary Figure S8).

In THP-1 cell line, a mate-pair analysis in chr1-chr20, chr9-chr11, and chr22-chr3 was performed to precisely locate the presence of split reads in translocated chromosomes. In t(20;1)(p11.22;q41), *ESRRG* gene was found at chromosome 1 while the non-coding region was detected at the corresponding chromosome 20; in t(9;11)(p22.3;q23.3), several split-reads were detected, *SNAPC3* gene was found in chromosome 9, and *KMT2A* gene was located at a corresponding region on the chromosome 11. In t(22;3)(q12.1;p21.23), two split reads were found, no coding gene was detected in chromosome 22, while *GSK3B* gene was located at the corresponding region in chromosome 3. Manta-based SVs results also supported the translocations detected by Hi-C data (Supplementary Figure S9).

### 3.4. PCR Amplification

**Figure 6:**
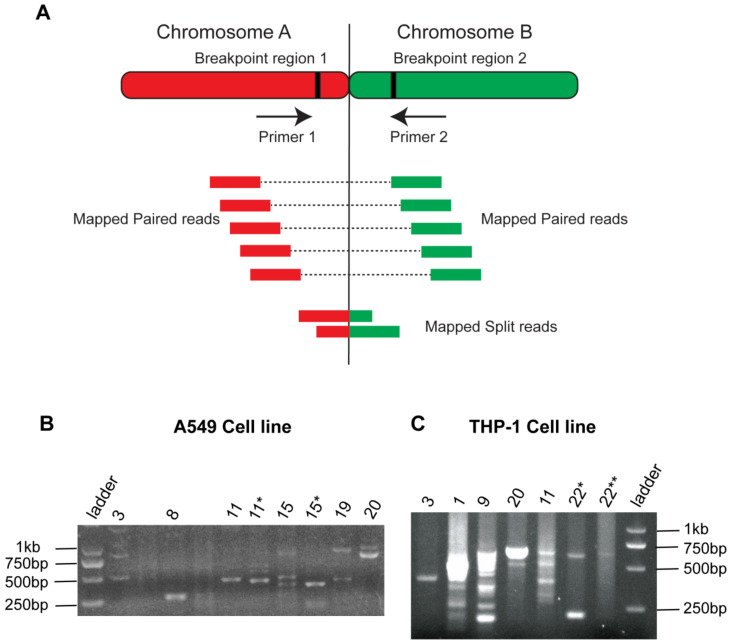
PCR Amplifications of translocated regions. (**A**) Illustration of primer design in the PCR validations method. (**B**) PCR validation results in A549 cell line (**C**) PCR validation results in THP-1 cell line. Gel bands represent the translocated reads of different sizes that appeared because of chromosomal translocations. * and ** indicate first and second replicates, respectively.

The common breakpoints between at least two tools were detected by Hi-C tools, extracted, and BLAST against the reference genome to find mapped paired reads. Forward and reverse primers were designed to amplify the mapped split reads (Supplementary Table S4), a detailed illustration of PCR validations is given in Figure 6A. Translocations (chr3-chr20, chr11-chr8, and chr15-chr19) in A459 and THP1 (3-22, 11-9, and 20-1) were successfully amplified, and multiple sized bands were observed (Figure 6). In lung cancer PCR results, the bands ranged from 250 bp to 2 kb were observed depending upon the read’s sizes. Most of the bands appeared in a size ranging from 500–750 bps. In the THP-1 sample, several PCR bands also showed the variable sizes of amplified reads ranging from 250 bp to 1 kb. Some of the bands appeared in the form of smear, which suggested that there might be a multi-sized read’s pool presence. In the K562 cell line, the reads from chromosome 3, 5, 6, 12, and 21 showed amplified band’s size in the range of 150–2000 bps. Some of the translocated reads showed no clear amplifications despite the repetitive efforts of primer designing (Supplementary Figure S10). The difficulty arises because the breakpoint regions might be present at the repetitive region or near the centromere. In both these conditions, PCR amplifications are challenging to obtain [32].

Overall, the integrative results have suggested that the translocation breakpoints identified using Hi-C data were validated by applying multiple data types/methods. Details of validations from each method are shown in Table 2.

## 4. Discussion and Conclusions

In the post-genomic era, next-generation sequence (NGS) technologies have made great revolutions in clinical human genetics. For example, whole-genome sequence (WGS) and genome-wide association studies (GWAS) have been used to replace traditional molecular diagnostics [33,34]. These techniques have been shown remarkable performance to outperform pre-existing single nucleotide variations (SNVs) detection methods such as whole-exome sequencing [35]. However, some drawbacks are associated with the usage of these techniques. They cannot properly detect and characterize the complex SVs such as translocations, duplications, insertions, and deletions, which significantly impact the three-dimensional organization by remodeling the genome [7]. Another limitation is the requirement of high sequencing depth to avoid the high rate of false positives of chromosomal rearrangements [36].

Hi-C data have proven more beneficial for detecting balanced chromosomal rearrangements than the standard sequencing methods, and it can detect the larger translocated blocks. Another advantage of Hi-C data usage is that it can provide an overview of chromosomal rearrangements in the Hi-C map; these larger translocated blocks also represent chromatin interaction frequency, which cannot be detected with pre-existing methods [37]. Several tools have been published to process Hi-C data and detect larger chromosomal rearrangements (translocations) [14,25,31]; each tool uses different biases for the detection of SVs. The results of each tool still need to be compared with pre-reported karyotypes and validated through an integrative approach. In this study, we have used Hi-C data, and WGS from different cell lines A549, K562, and THP-1, and processed through HiC_trans, HiNT-TL, and hic_breakfinder tools to find complex chromosomal rearrangements. Additionally, PCR amplification of translocation breakpoint regions, and mate-paired regions for each cell line were performed.

In A549 cell line, we identified four chromosomal pairs that undergo translocations such as chr3-chr3, chr3-chr20, chr15-chr18, and chr9-chr11. Both HiC_trans and hic_breakfinder detected above mentioned chromosomal translocations; however, HiNT-TL detected translocations only in the chr8-chr11 pair. Most of the translocations were novel results, with one exception—translocation t(15;19)(q11;p13) was previously reported in a FISH karyotype study of lung cancer patients. Chromosome 15 translocations were previously reported to be involved in the overexpression of the *Notch3* gene in a female. Immunological studies have suggested the presence of poorly differentiated epithelial cells [38]. The functional studies of novel translocations in the A549 cell line need to be explored by further experiments. Several protein-coding genes such as *ROBO2*, *FAT3*, and *ZNF43* were detected at the breakpoint regions. Previously reported studies have suggested the direct or indirect regulatory involvement of these genes in lung cancer development [39,40,41]. In K562 cell line, a total five inter-chromosomal translocations were detected (chr15-chr1, chr3-chr10, chr6-chr5, chr12-chr21, and chr6-chr16). Some of the translocations were previously reported and well-characterized in leukemia cancer [31,42]. In t(3,10)(p21.33;q23.2), two protein-coding genes, i.e., *CDG25A* and *GRID1* were detected. The previously reported study from Cancer Genome Atlas Research Network suggested the *GRID1* gene fusion in chronic myelogenous leukemia (CML). *GUSBP1* (pseudogene) was also detected at the translocation site in chromosome 5. In the THP-1 (acute monocytic leukemia) cell line, three chromosomal translocations were detected (chr1-chr20, chr22-chr3, and chr11-chr9), which were previously reported by a FISH study [20]. However, the exact details about breakpoint locations were not available previously. Overall, Hi-C detection provides more sensitive details about the translocations at the highest resolution.

Previously, WGS technology has been used to detect translocations, so we cross-validated the results of Hi-C with translocations detected through WGS data. The results of both technologies were coherent, which suggested the high confidence in Hi-C findings. Several published studies have suggested that translocations may have several functional impacts on the gene; one of the significant impacts is the “gene fusion” [43]. This gene fusion mechanism has drastic effects on the expression of genes involved to cause cancer progression, *PML-RAR* [44] and *BCR-ABL1* [45] are the classic examples.

Furthermore, PCR analyses for all samples were performed; results suggested that translocated pairs showed clear gel bands in A459 and THP-1 cell lines. Gel bands showed the presence of translocated split reads. Some of the translocations in K562 were not amplified/appeared as a smear, which might be because of the complex rearrangements. For example, the translocations are followed by the deletions, which result in the removal of primers annealing sites, or are due to the large fragments insertions which made that particular region too long to be amplified [32].

Overall HiC_trans detected a larger number of translocations comparatively, at multiple intermediate lower resolutions. Visual inspections of HiC_trans results suggested the presence of a high false-positive rate. Hic_breakfinder detects a relatively smaller number of translocations at the highest resolutions, while HiNT-TL detects fewer translocations. The difference in detection power is quite evident because all these tools use different strategies. Upon validations through WGS, we have observed that hic_breakfinder results were more overlapped with WGS than others.

In summary, our study indicates that Hi-C data are reliable for identifying and characterizing large complex translocations at a sequence and 3D genome structure level. Among pre-existing tools, hic_breakfinder detects more reliable translocation breakpoints, which can be utilized for downstream analysis. Our data showed that Hi-C-based results are no less helpful than WGS. PCR amplification results also supported the findings of Hi-C, which put more confidence to add Hi-C technology as a suitable assistive method for translocation detection in cancer genomes. The addition of Hi-C technology with other tools will be helpful to obtain deep insight into the detection and characterization of complex rearrangements that cause pathogenic phenotypes.

## Figures and Tables

**Table 1:** Detailed summary of the datasets used for analyses. The following table gives the accession number, cell line, and datatypes of the samples used in this study.

Cell Line	Exp.ID/Accession	Data Type	Data Availability
A549	Lab data	In situ Hi-C_rep1	*In-house*
A549	Lab data	In situ Hi-C_rep2	*In-house*
A549	SRR14843382	WGS	Public
K562	SRR6468005	*DLO* Hi-C_rep1	Public
K562	SRR6468006	*DLO* Hi-C_rep2	Public
K562	SRR14828339	WGS	Public
THP-1	SRR5005053	DLO Hi-C	Public
THP-1	SRR8670675	WGS	Public

**Table 2:** Integrative summary of translocation breakpoints and cross-validation results.

Cell Line	Hi-C Breakpoints	WGS Breakpoints	PCR
A549	Chr11:92110000-92340000 Chr8:125920000-125950000	Chr11:92232864-92337991 Chr8:125915486-125920628	√
A549	Chr3:69600000-76000000 Chr20:24900000-26500000	Chr3:759889069-75989055 Chr20:26204286-26204887	√
A549	Chr15:22298000-22368000 Chr19:22005000-22019000	Chr15:22290005-22291154 Chr19:22004010-22005005	√
K562	Chr1:34540000-34750000 Chr15:79000000-79220000	Chr1:164354987-164355279 Chr15:79163087-79163379	×
K562	Chr3:48257000-48287000 Chr10:87878000-87989000	Chr3:48227444-48228179 Chr10:87848597-87849236	√
K562	Chr5:21560000-21575000 Chr6:57680000-57760000	Chr5:21573105-21573417 Chr6:57575670-57575919	√
K562	Chr12:22560000-22566000 Chr21:25532000-25534001	Chr12:22711223-22711519 Chr21:25504958-25505270	√
K562	Chr6:16753000-16768000 Chr16:85549000-85567000	Chr6:33618899-33619146 Chr16:63908450-63908595	×
THP-1	Chr9:14520000-15460000 Chr11:118350000-118900000	Chr9:15458220-15458580 Chr11:118356100-118356400	√
THP-1	Chr22: 29020000-29060000 Chr3: 119580000-119670000	Chr22:29081888-29081988 Chr3:119651343-119651367	√

## Supplementary Materials

The following are available online at https://www.mdpi.com/article/10.3390/genes13071170/s1, Figure S1: Differential Rearrangements at the chromosomal level in K562 cell line. Figure S2: Differential Rearrangements at the chromosomal level in THP-1 cell line. Figure S3: Translocations identified in K562 cell line. Figure S4: K562 translocations result from comparison between tools. Figure S5: Breakpoints identified in THP-1 Hi-C data. Figure S6: THP-1 translocations results from comparison between tools. Figure S7: Mate-pairs analysis of A562 WGS data. Figure S8: Mate-pairs analysis of K562 WGS data. Figure S9: Mate-pairs analysis of THP-1 WGS data. Figure S10: PCR-Amplification of K562 breakpoint region. Table S1: Hi-C Trans Result in A549. Table S2: hic_breakfinder in A459 Cell line. Table S3: HiNT-TL in A459 Cell line. Table S4: PCR primer designed for translocation amplifications.
