# Supplementary material for: Chromosomal Translocations Detection in Cancer Cells Using Chromosomal Conformation Capture Data
Source: Genes (Basel). 2022 Jun 29;13(7):1170. doi: 10.3390/genes13071170 (PMC9319866; doi:10.3390/genes13071170)
Supplement: Supplementary file 1 [file genes-13-01170-s001.zip › Supplementary Figures.pdf]

Supplementary Figures

Chromosomal Translocations Detection in Cancer Cells Using Chromosomal Conformation Capture Data

Muhammad Muzammal Adeel[1,2,3], Khaista Rehman[4,5,6], Yan Zhang[1], Yibeltal Arega[7], Guoliang Li[1,2*]

[1]National Key Laboratory of Crop Genetic Improvement, Huazhong Agricultural University, Wuhan, Hubei, 430070, China

[2]Agricultural Bioinformatics Key Laboratory of Hubei Province, Hubei Engineering Technology Research Center of Agricultural Big Data, 3D Genomics Research Center, College of Informatics, Huazhong Agricultural University, Wuhan, Hubei, 430070, China

[3]Department of Environmental Health Science, College of Public Health, University of Georgia Athens, GA 30602, USA

[4]State Key Laboratory of Agricultural Microbiology, Huazhong Agricultural University, Wuhan, Hubei, 430070, China

[5]College of Veterinary Medicine, Huazhong Agricultural University, Wuhan, China

[6]College of Bio-Medicine and Health, Huazhong Agricultural University, Wuhan, China

[7]Departments of Population Health & Environmental Medicine, New York University 180 Madison Ave New York, NY 10016, USA

***Correspondence:**

Guoliang Li
guoliang.li@mail.hzau.edu.cn

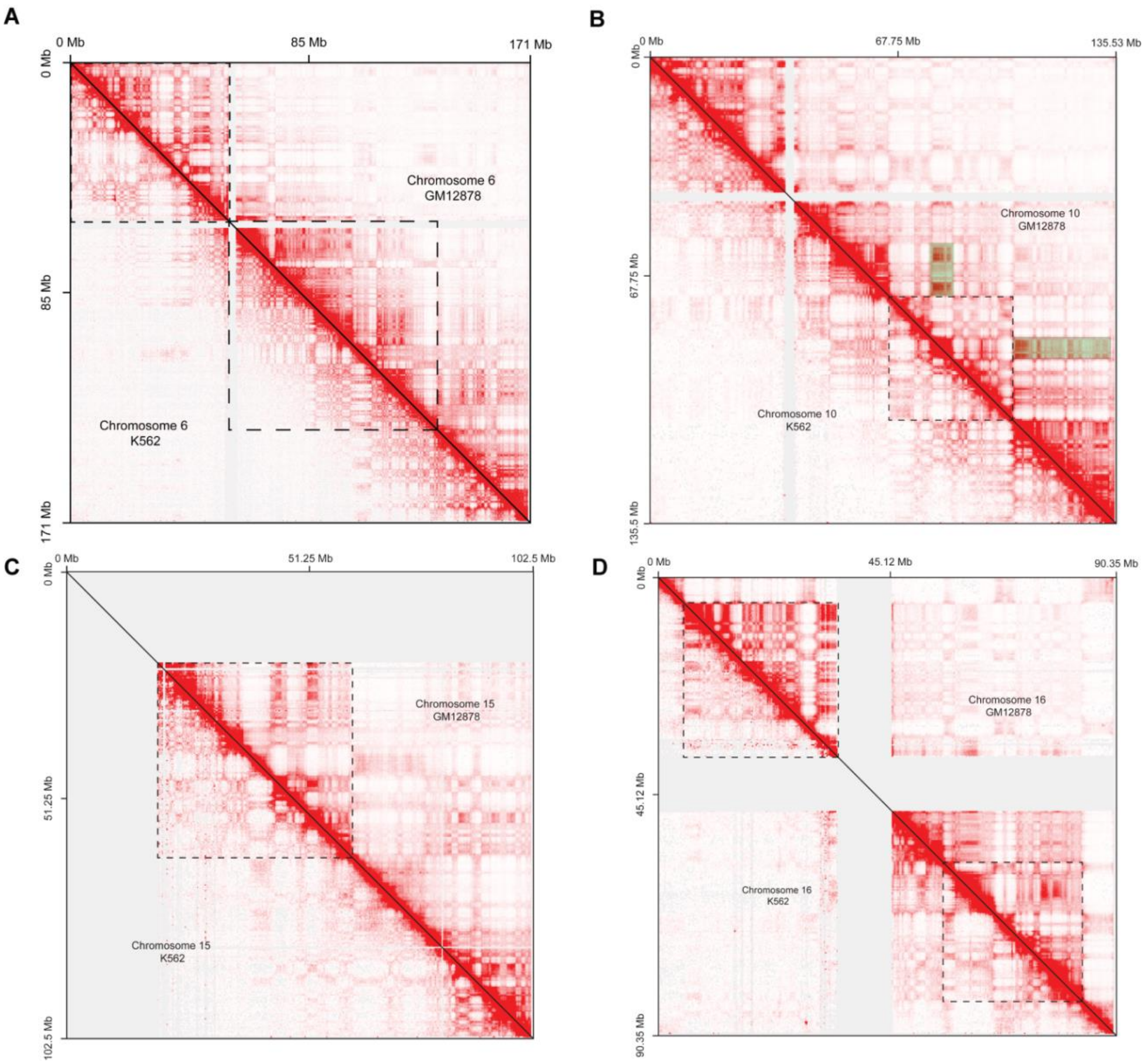

Figure S1. Differential Rearrangements at the chromosomal level in K562 cell line. Structurally variable regions are shown at a chromosomal level, **(A)** chromosome 6, **(B)** chromosome 10, **(C)** chromosome 15 and, **(D)** chromosome 16. Dotted lined boxes represent changes.

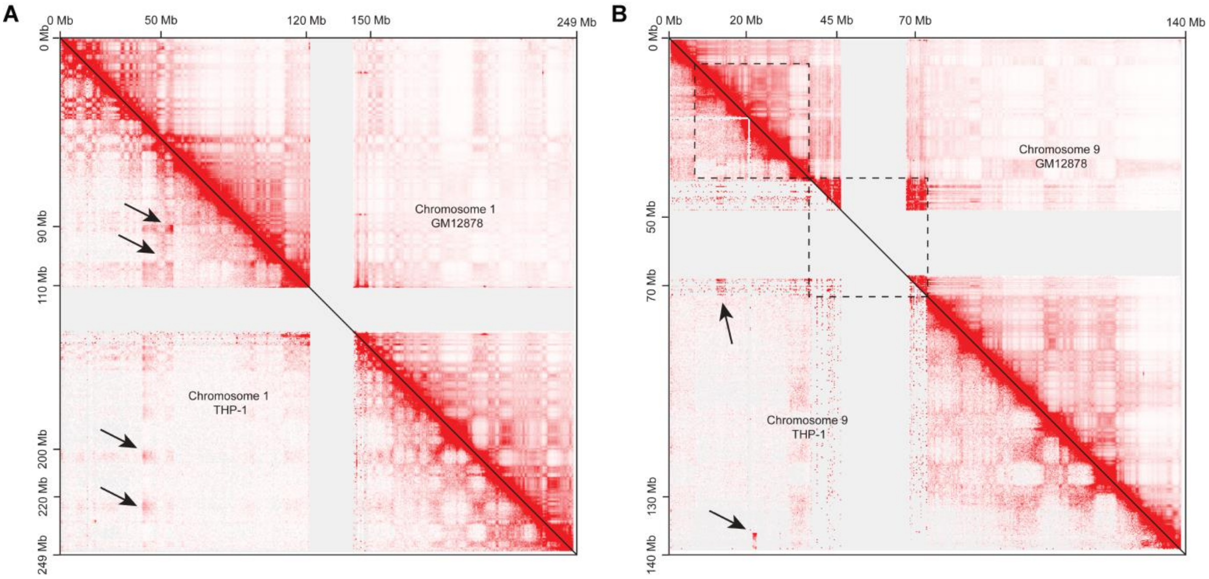

Figure S2. Differential Rearrangements at the chromosomal level in THP-1 cell line (A) Chromosome 1, and **(B)** Chromosome 9. Differential regions were indicated by black arrow-head (left) and black dotted boxes (right).

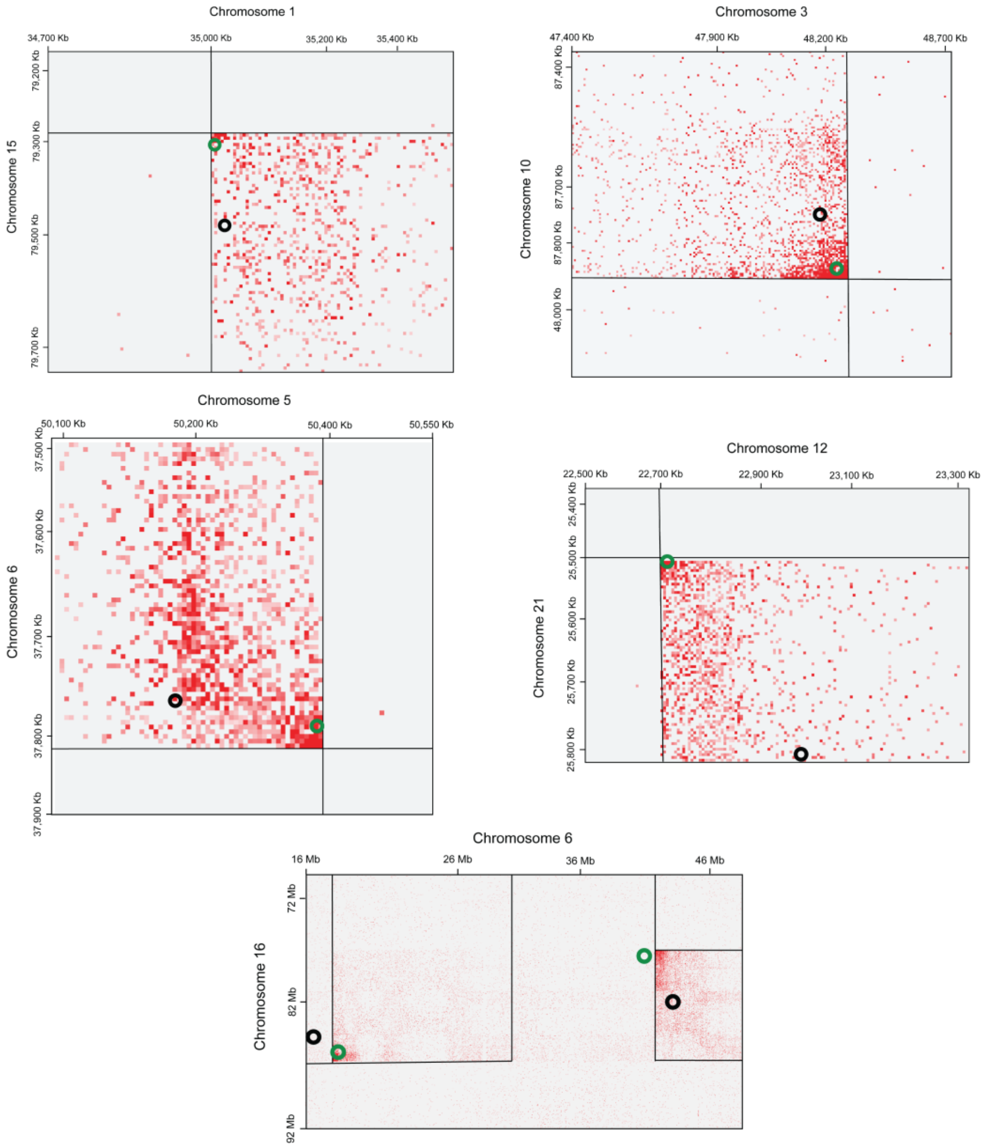

Figure S3. Translocations identified in K562 cell line Several breakpoints are shown in chr1-chr15, chr3-chr10, chr5-chr6, chr12-chr21, and chr6-16. Probable locations were indicated with the circles. The green circle shows the results detected by hic_breakfinder, while the black circle represents results from HiCtrans.

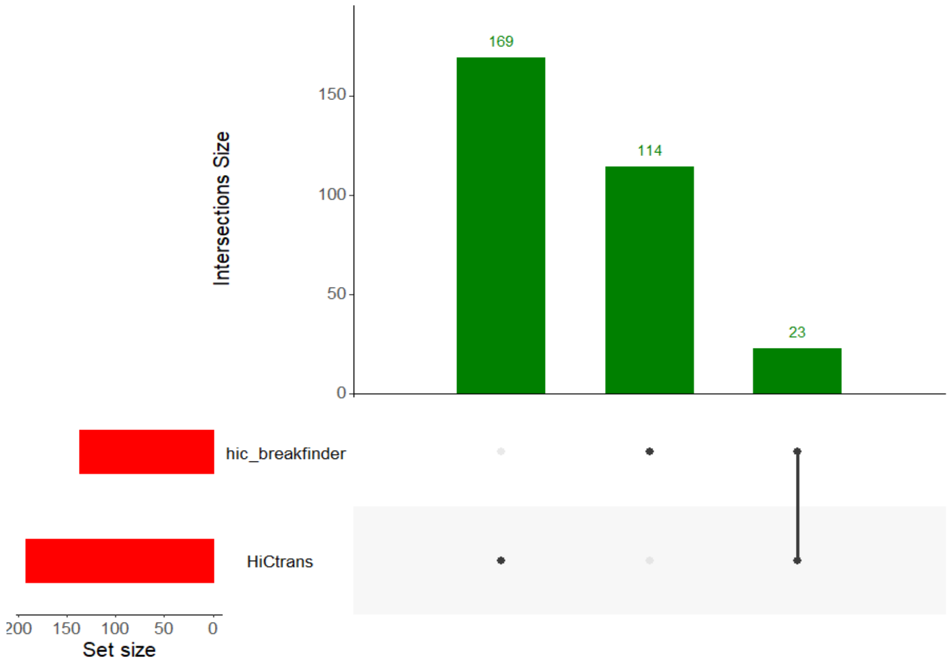

Figure S4. K562 translocations result from comparison between tools The upSet plot of breakpoints results detected by different methods. The bottom left panel red horizontal bars represent the number of translocations detected by each method; vertical green bars represent the breakpoint intersections of each translocation set. Black dots show the sample set, and a vertical black line represents the intersection between methods. This graph is generated by UpSet R package.

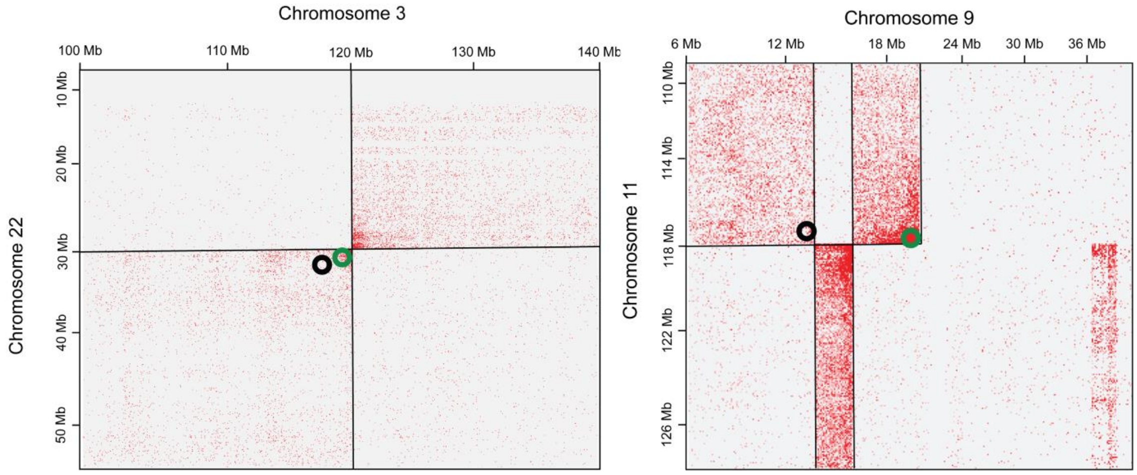

Figure S5. Breakpoints identified in THP-1 Hi-C data Several breakpoints are shown in chr3-chr22 and chr9-chr11. Breakpoint locations were indicated with the circles. The green circle shows the results detected by hic_breakfinder, while the black circle represents results from HiCtrans.

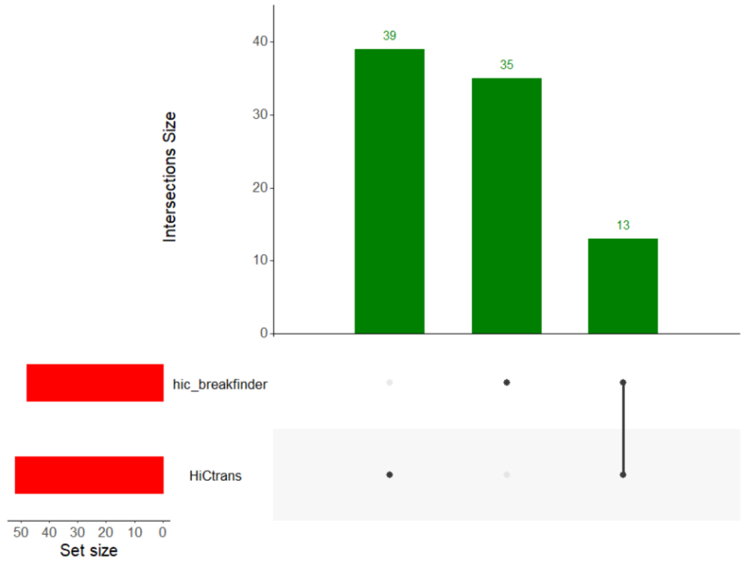

Figure S6. THP-1 translocations results from comparison between tools The UpSet plot of breakpoints results detected by different methods. The bottom left panel red horizontal bars represent the number of translocations detected by each method; vertical green bars represent the breakpoint intersections of each translocation set. Black dots show the sample set, and a vertical black line represents the intersection between methods. This graph is generated by UpSet R package.

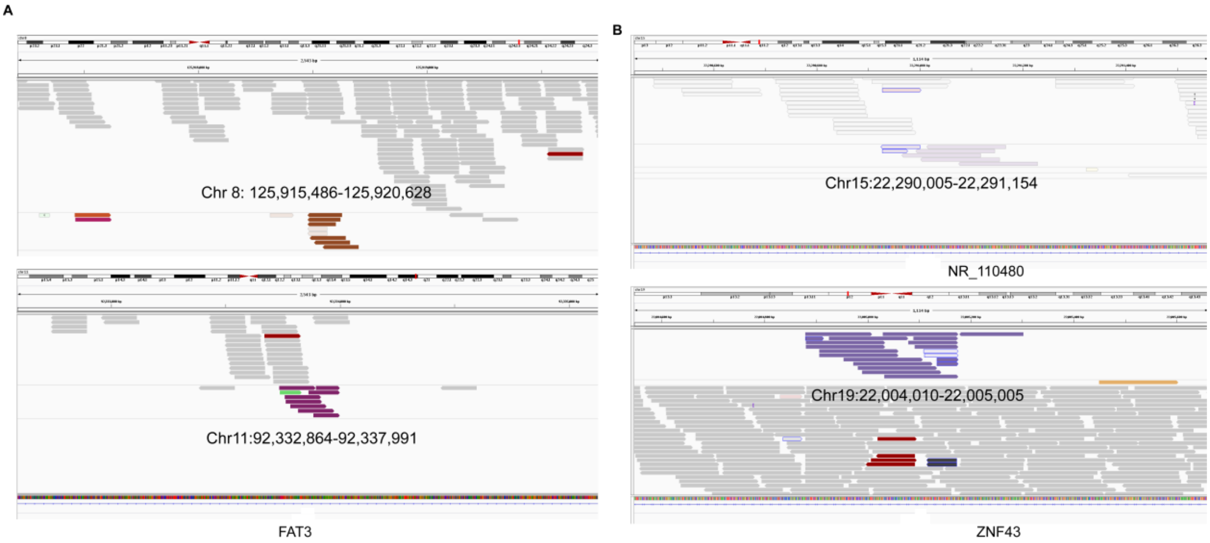

Figure S7. Mate-pairs analysis of A562 WGS data The translocation events in A549 WGS data are visualized by the IGV tool, showing WGS paired reads supporting the translocation results of Hi-C data. **(A)** In t(8;11)(q24.13;q14.3), the upper panel represents the reads from chr8 in dark brown color, while in the lower panel, corresponding mate pairs in chr11 are highlighted with purple color. **(B)** In t(15;19)(q11.2;p12.0) translocation mate-pairs reads are represented with light grey (upper panel) and light-purple color (lower panel).

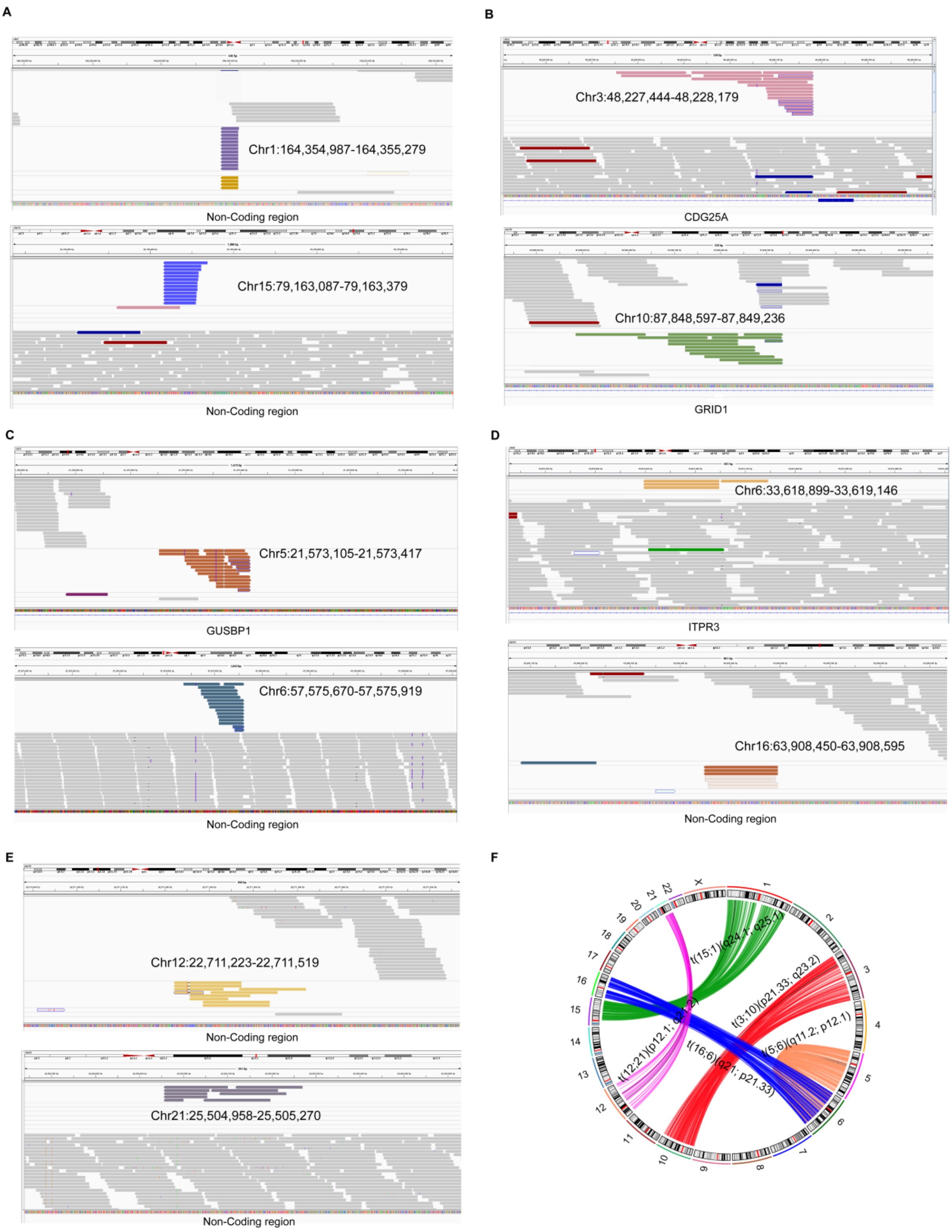

Figure S8. Mate-pairs analysis of K562 WGS data The translocation events in K562 WGS data are visualized by the IGV tool, showing WGS paired reads supporting the translocation results of Hi-C data. **(A)** In t(15;1)(q24.1;q25.1) upper panel representing the reads from chr1 in light purple color, while in the lower panel, corresponding mate pairs in chr15 are highlighted with blue color. **(B)** In t(3;10)(p21.33;q23.2), mate pairs are shown in upper and lower panel with deep pink and light-green

color, respectively, and **(C)** In translocation t(5;6)(q11.2;p12.1) mate-pairs reads are represented with deep-brown (upper panel) and deep-blue color (lower panel). **(D)** t(16;6)(q21;p21.33) mate-pairs are shown with deep-brown (lower panel) and yellow color in upper panel. **(E)** t(12;21)(p12.1;q21.2) mate pairs are highlighted with yellow color in upper panel and grey color in lower panel. **(F)** In the circos plot, translocations detected by Manta in each chromosomal pair are shown with red, orange, blue, pink, and green colors. Hi-C overlapped results were highlighted with black arcs.

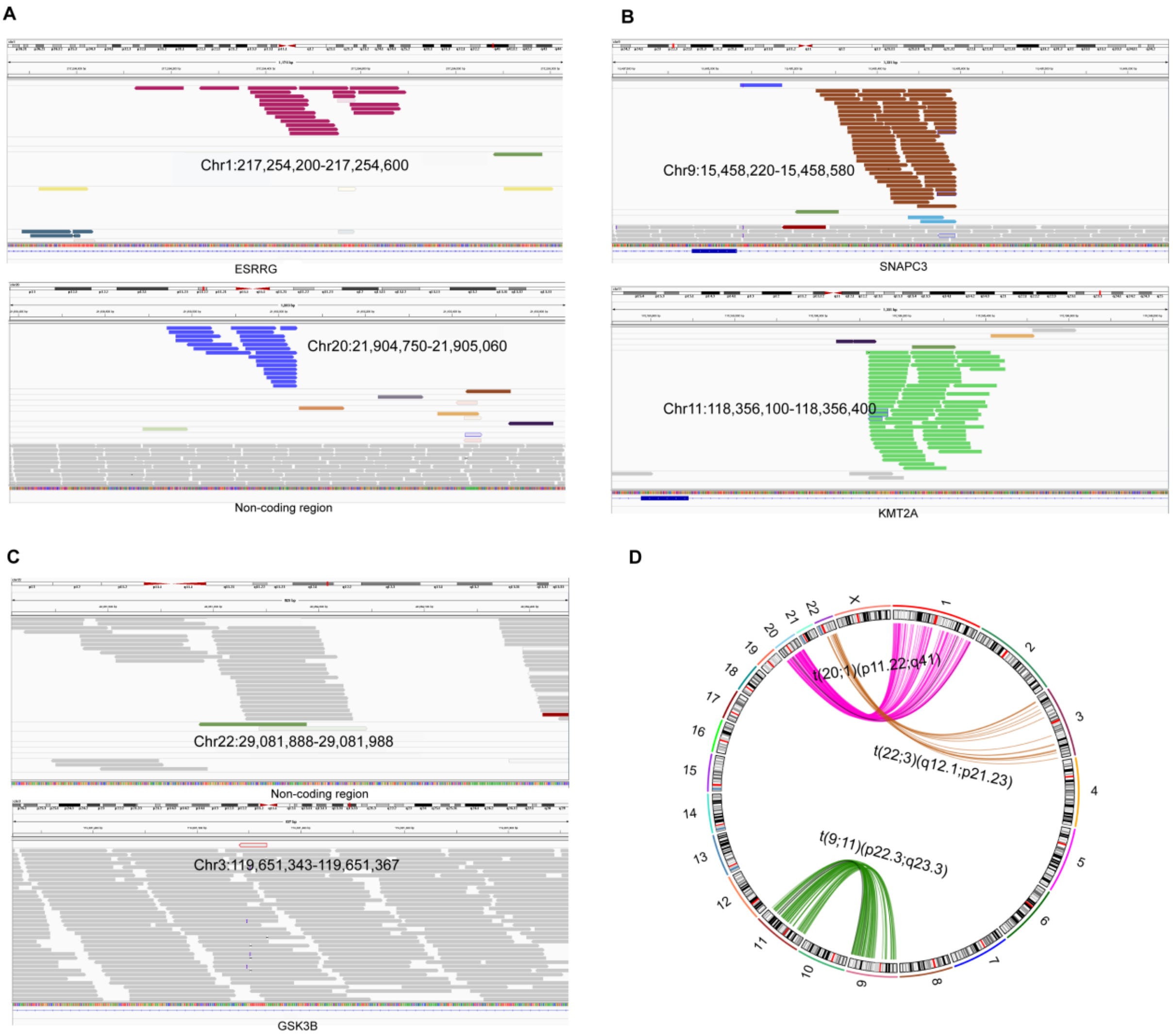

Figure S9. Mate-pairs analysis of THP-1 WGS data The translocation events in THP-1 WGS data are visualized by the IGV tool, showing WGS paired reads supporting the translocation results of Hi-C data. **(A)** In t(20;1)(p11.22;q41), the upper panel is representing the reads from chr1 in deep magenta color, while in the lower panel, corresponding mate pairs in chr20 are highlighted with deep blue color. **(B)** In t(9;11)(p22.3;q23.3) , mate pairs are shown in upper and lower panel with deep brown and light-green color, respectively, and **(C)** In t(22;3)(q12.1;p21.23) translocation mate-pairs reads are represented with green (upper panel) and light-grey color (lower panel). **(D)** In circos plot, translocations detected by Manta in each chromosomal pair are shown with pink, brown, and green. Hi-C overlapped results were highlighted with black arcs.

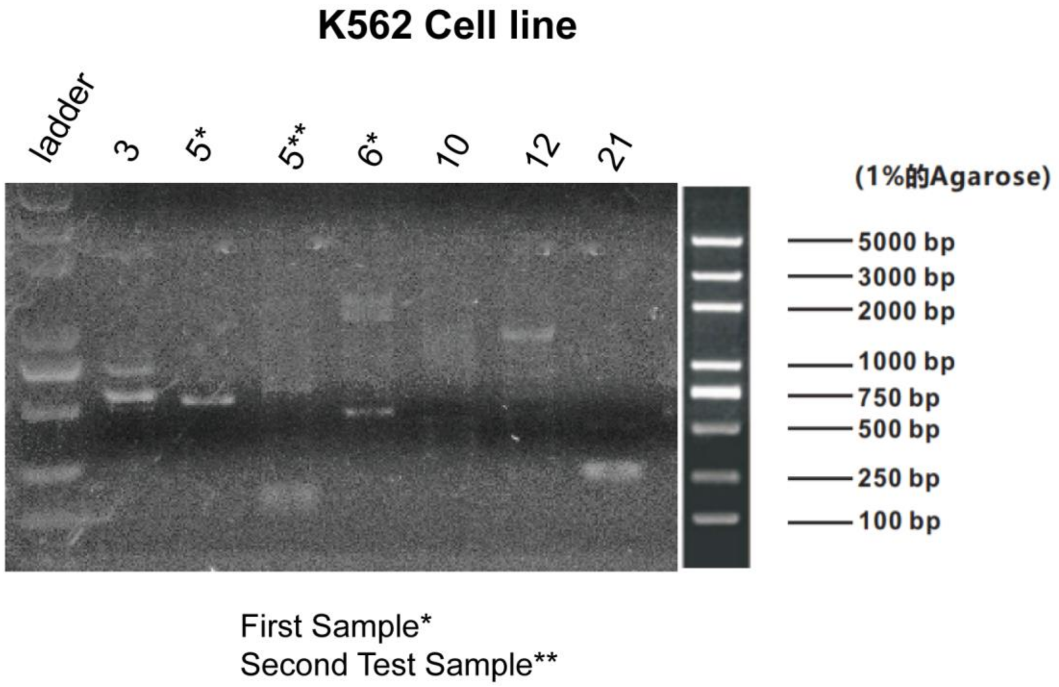

Figure S10. PCR-Amplification of K562 breakpoint region. Upper panel on the gel representing the chromosome number.
